# Using Life Course Theory to Explore the Association Between Autistic Traits, Child, Family, and School Factors and the Successful Transition to Secondary School

**DOI:** 10.1007/s10803-022-05845-z

**Published:** 2023-04-06

**Authors:** Moira Whelan, Jane McGillivray, Nicole J. Rinehart

**Affiliations:** 1 Deakin Child Study Centre, School of Psychology, Deakin University, Geelong, VIC Australia; 2 School of Educational Psychology and Counselling, Faculty of Education, Monash University, Clayton, VIC Australia

**Keywords:** Autistic traits, School transition, Mental health, Life Course Theory, Quality of Life, School belonging

## Abstract

Life Course Theory contends that school transitions can interrupt academic and wellbeing trajectories, depending on child, family, and school factors. Hierarchical regression analyses examined how autistic traits were associated with school transition outcomes. Autistic traits explained 12% of the variance in Quality of Life (QOL), 24% of the variance in mental health and 9% of the variance in school belonging. When autistic traits were accounted for, gender was a significant predictor of changes in QOL whereas changes in school belonging were predicted by cognitive functioning, parent education, school attendance and school refusal. Changes in mental health after transition were mostly predicted by family factors including family structure, family functioning and parent education but were also significantly predicted by sleep problems.

## Using Life Course Theory to Explore the Association Between Autistic Traits, Child, Family, and School Factors and the Successful Transition to Secondary School

The transition to secondary school is an important educational milestone which can adversely impact school belonging, mental health and academic progress (Anderson et al., 2000; Evans et al., 2018; Symonds & Galton, 2014). According to Life Course Theory, transitions intersect developmental trajectories with the potential to act as positive or negative disruptors depending on child, family and school factors as well as the broader ecological context in which the transition occurs (Benner, 2011). The Life Course perspective views physical, cognitive and psychological developmental pathways as interconnected and provides an organising framework to study the various domains that describe successful school transition and to make connections between them (Benner, 2011). These include academic progress, social integration and emotional wellbeing (Anderson et al., 2000; Evans et al., 2018).

Students with autism are thought to be vulnerable during school transition as they often find change aversive and experience social communication and social interaction difficulties and sensory challenges which can hinder successful transitions (Mandy et al., 2016; Nuske et al., 2019). Three recent systematic reviews of school transitions for students with autism have provided a comprehensive picture of the key foci of the transition literature (Nuske et al., 2019; Richter et al., 2019; Stack et al., 2021). Most studies employed qualitative designs, focussing on the experience of transition from various stakeholder’s perspectives and the type of transition supports offered (Dann, 2011; Dillon & Underwood, 2012; Stack et al., 2021). Several qualitative accounts of school transition for autistic^1^ students described students struggling to adapt to changing social, organisational and academic environments (Dillon & Underwood, 2012; Peters & Brooks, 2016). Yet not all students described difficulties and some were reportedly flourishing at secondary school (Neal & Frederickson, 2016) with no clear understanding of why some autistic students were faring better than others. Fewer studies used quantitative measures and these studies only focused on one or two domains of functioning. Mandy and colleagues (2016) examined adaptive functioning, mental health and peer victimisation. Fortuna (2014) examined social and emotional functioning, Hebron (2018) examined school belonging and Hannah and Topping (2012) examined anxiety. The common finding in these studies was that students varied widely in their functioning across transition however there was no evidence of an escalation of problems at secondary school.

One possible explanation for the variable transition outcomes in both the qualitative and quantitative literature is that these studies treated autistic students as one homogenous group with no measurement of autism symptom severity. If autistic traits exist on a spectrum (as the condition’s title denotes), at what point do these autistic traits interfere with school functioning? Moreover, there is accumulating evidence that subclinical autistic traits are associated with poorer academic performance, behavioural problems, negative attitudes to school and problematic relationships with peers and teachers (Hsiao et al., 2013; Skuse et al., 2009) therefore it is important to examine how autistic traits are associated with transition outcomes. Accordingly, a recent study examined how autistic traits in a school-based sample of students were associated with various indicators of transition success (Whelan et al., 2021). Unexpectedly, autistic traits were associated with improved Quality of Life (QOL) and mental health after school transition which was conjectured to result from a preference for the secondary school environment and to the positive benefits of a “clean slate” after previous negative experiences in primary school. It is also possible that other child, family and school factors were contributing to these positive findings and Life Course theory emphasises how individual characteristics, resources, and experiences influence school transition along with family, school and wider sociocultural contexts (Benner, 2011). Yet only one previous study was identified which assessed child and school factors associated with school transition for autistic students (Makin et al., 2017). It examined cognitive function, autism severity, sensory difficulties, and anxiety in a sample of fifteen students with ASD (only seven of whom transferred to a mainstream secondary school). None of these child factors predicted transition success although the small sample size warrants further investigation of how these child factors influence transition outcomes. The study reported that it was predominately school factors that influenced school transition success such as poor communication between schools and delays in school placements (Makin et al., 2017).

Many factors have been identified in the wider (non-autistic) school transition literature that may render students more vulnerable during transition. They include sociodemographic variables such as age (Galton et al., 2000), gender (Anderson et al., 2000; Symonds & Galton, 2014) and family SES (Akos et al., 2014); child factors such as internalising and externalising problems and/or challenging behaviour (Palmu et al., 2018; Riglin et al., 2013) and lower academic ability (West et al., 2010); family factors such as parental involvement (Duineveld et al., 2017; Waters et al., 2014) and family cohesion and adaptability (Oriol et al., 2017; Symonds & Galton, 2014); and school factors such as school SES (Chesters, 2019; Perry & McConney, 2010), school attendance (Hancock et al., 2013) and school refusal (Gonzálvez et al., 2020; Kearney, 2008). It is difficult to isolate the influence of individual contributors to transition success, and it is likely that they interact in complex ways (Jindal-Snape et al., 2020).

Life Course Theory also underscores the importance of examining the sociocultural contexts in which transitions occur. This research took place at a time when school inclusion was a priority at all levels of government both in Australia and internationally (Office of the United Nations Hight Commissioner for Human Rights (OHCR), 2013). The meaning of inclusion has evolved from being physically present in a mainstream classroom to encompass positive learning, a sense of belonging and wellbeing at school (Merry, 2020). Yet many autistic students experience “exclusion within inclusion” (Williams et al., 2019) and there is considerable qualitative research to support the contention that mainstream education is challenging for students with autism (Goodall, 2020; Saggers, 2015). This research also took place during a time of rapid change in how autism is described and understood (Kenny et al., 2016). These changing landscapes influence school policies, teacher attitudes and practices, social relationships at school and even the way autistic students view their own identity (Williams et al., 2019).

Although studies examining transition with autistic students typically focus on just one domain of school functioning (Nuske et al., 2019), successful transition is a multidimensional construct encompassing academic, social and emotional wellbeing domains (Evangelou et al., 2008; Evans et al., 2018). Accordingly, this study examined changes in three indicators of social-emotional wellbeing: Quality of Life (QOL), school belonging and mental health. It is increasingly recognised that mental health is more than the absence of negative symptoms (Manwell et al., 2015). Assessments of positive mental states, therefore, are increasingly valued in health research (Berman et al., 2016). QOL has been defined as “an individual’s perception of life in the context of the culture and value systems in which they live and in relation to their goals, expectations, standards, and concerns” (World Health Organization, 1995, p. 1405). It provides information on functioning in the home and at school and can be used as an indirect measurement of mental health status (Berman et al., 2016). QOL has previously been used as a gauge of transition success in children with and without disability (Gillison et al., 2008; Jones et al., 2009) and is considered the gold standard for measuring wellbeing in individuals with autism (McConachie et al., 2015). School belonging is a measure of a student’s social adaptation at school (Benner & Graham, 2009) and is, therefore, an important indicator of successful transition. School belonging refers to a student’s perception of “psychological membership in the school or classroom” and is defined as “the extent to which students feel personally accepted, respected, included, and supported by others … in the school social environment” (Goodenow & Grady, 1993, pp. 60–61). In a recent meta-analysis of 82 studies, school belonging was found to play an important role in students’ success and wellbeing, being positively associated with many favourable school outcomes such as academic achievement, social-emotional health and school engagement (Korpershoek et al., 2020). Lower perceptions of school belonging, however, have been associated with disengagement, school refusal and drop out (Anderson et al., 2000; Fine, 1991, as cited in Ma, 2003).

Mental health is the third indicator of successful school transition in this study due to its association with many important school outcomes (Benner et al., 2017; Riglin et al., 2013). For example, an Australian study examining the relationship between child and adolescent mental and educational outcomes found that students with mental health conditions scored lower on standardised tests in each academic domain at each year level tested, had lower school attendance, lower school engagement and school connectedness (Goodsell et al., 2017). The mental health of students during school transition is also an important focus as studies attest to the increase in anxiety, depressive symptoms and loneliness during this time (Benner et al., 2017). In Australia, a national survey of the mental health and wellbeing of children and adolescents reported that one in seven students met the Diagnostic and Statistical Manual of Mental Disorders Version IV (DSM-IV) criteria for a mental disorder in the previous 12 months (Lawrence et al., 2015) and more than half of mental disorders had their onset before the age of 14 (Kessler et al., 2007).

The aim of the current study was to examine how autistic traits were associated with three indicators of transition success and to examine if child, family and school factors explained additional variance in these transition outcomes once autistic traits were accounted for. It was hypothesised that autistic traits would be associated with lower QOL, mental health, and school belonging after the school transition, however child, family and school factors were hypothesised to be significantly associated with transition outcomes and to explain additional variance once autistic traits were controlled for.

## Methods

### Study Design and Setting

This study was a prospective observational study of a school-based sample of students during the transition from primary to secondary school. All mainstream government, catholic and independent schools in a regional city were invited to participate. The city has a population of 278,929 as of the 2016 census. 68% of primary school children attend government schools, 21% attend catholic primary schools, and 11% are educated in the independent sector.

### Participants

Fifty-one students in their final year of mainstream primary school (Grade 6) with a mean age of 12.2 years (SD = 4.8) participated in this study. Table 1 presents the student and family characteristics of the study participants.

**Table 1:** *Sample Characteristics*

Characteristics	
Child Age (M, SD, range)	12.15 (4.8, 11.33–13.5)
Male, n (%)	29.00 (56.9)
IQ, FSIQ^a^ (M, SD, range)	108.28 (11.51, 88–133)
Autistic traits^b^ (M, SD, range)	34.52 (30.65, 4-127)
ADHD Inattention traits^c^	-6.10 (12.06)
ADHD hyperactivity traits^c^	-7.98 (11.72)
ODD traits^c^	-4.83 (10.26)
Anxiety Symptoms^d^	14.49 (9.66)
Sleep problems^e^	45.20 (4.98)
Family Socio economic status (SEIFA)^f^	1019 (57)
Living with both parents, n (%)	33 (78.57)
Living with one parent, n (%)	2 (4.76)
Living with parent & new partner, n (%)	4 (9.52)
Living with other family member, n (%)	3 (7.14)
Mother’s education Post-graduate	16 (39)
Bachelor	9 (22)
Certificate/Diploma	12 (29.3)
Year 12 or equivalent	4 (9.7)
Mother’s employment Full-time	10 (24.4)
- Part-time	24 (58.5)
- Stay at home parent	6 (14.6)
- Student	1 (2.4)

^a^full scale IQ on 4 subsets (Block Design, Vocabulary, Matrix Reasoning, Similarities) of the WASI-II.

^b^Social Responsiveness Scale – 2nd edition

^c^The SWAN rating scale for ADHD & ODD. Negative scores represent a strength

^d^SCAS anxiety scale

^e^Children’s Sleep Habits Questionnaire (CSHQ)

^f^Socio-Economic Indexes for Areas (M = 1000, SD = 10)

### Procedure

The study was approved by the University’s Human Research Ethics Committee, the Department of Education and Training and Catholic Education. Three independent schools reviewed the request to conduct research at their schools and received approval by the School Board.

#### Study Recruitment

**Fig. 1:**
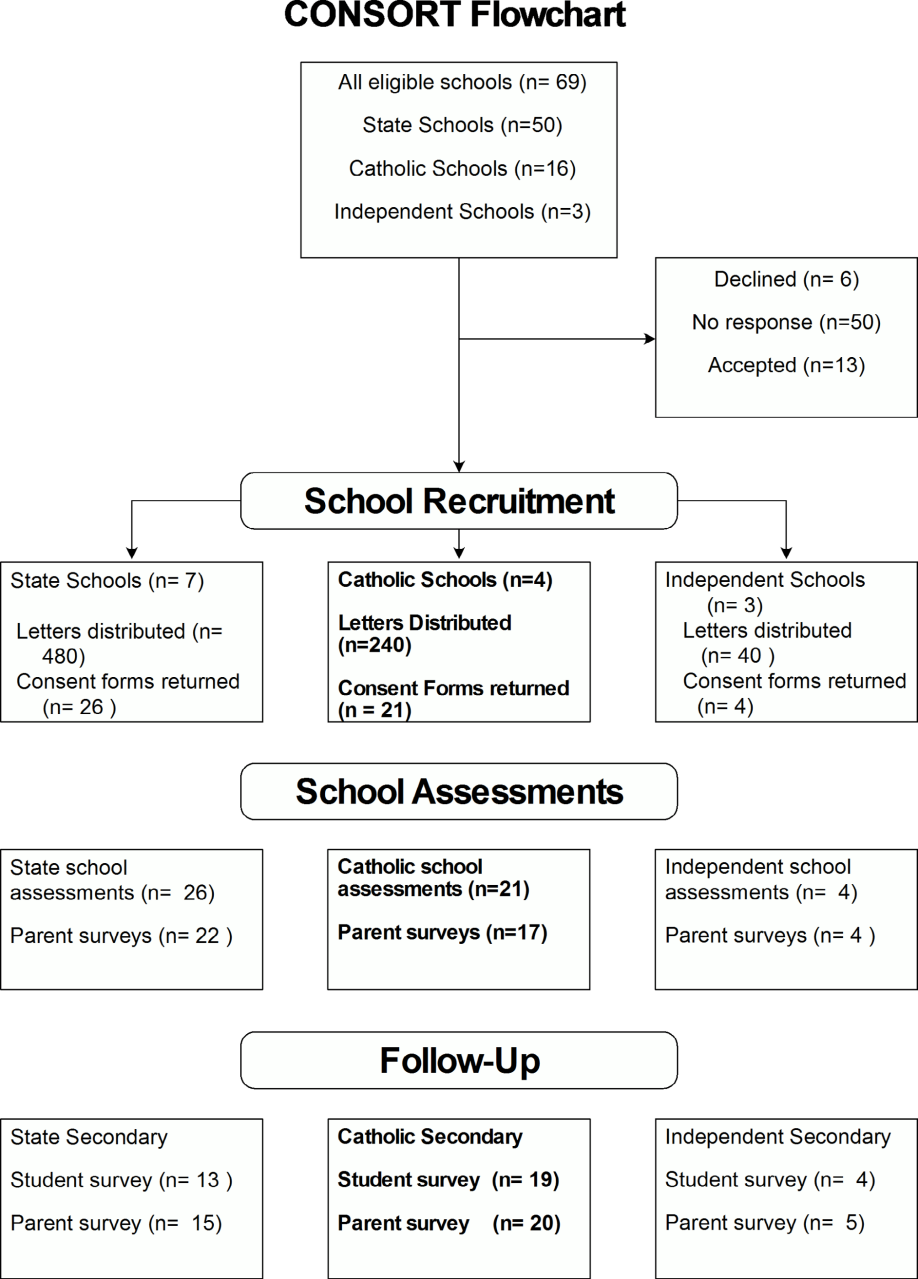
**top** *School Recruitment Process*

An email invitation was sent to the principal of every mainstream primary school in the region (n = 69). Nine schools responded to the email (5 accepting and 4 declining). Sixty hard copy invitations were then posted to the remaining schools. Eight schools returned a consent form and there was no response from the other schools. No further contact was made to these non-responsive schools in recognition that they receive an abundance of requests for research each year. Each school that provided a consent form received recruitment packs to be distributed to their Grade 6 students consisting of an explanatory letter, Plain Language Statement, consent forms for parents and for students, and a reply-paid envelope. Figure 1 details the school recruitment process.

#### Data Collection – Pre-transition

School visits commenced in August (3rd term) and continued through to December. Each student received two school visits, one week apart, lasting approximately 45–50 min. All students worked one-on-one with one author (MW) in a quiet room nominated by the school principal. Data from parents was collected via online surveys (or paper surveys if requested) from August until December.

#### Student Assessments – Pre-transition

Students completed all four subsets of the Wechsler Abbreviated Scale of Intelligence™ - second edition (WASI-II): Block Design, Vocabulary, Matrix Reasoning, and Similarities. All assessments were conducted in the same order and in accordance with the WASI-II training manual. Students also completed four paper-based questionnaires: the KIDSCREEN 27, the Strengths and Difficulties Questionnaire, the Psychological Sense of School Membership (PSSM), and the Spence Anxiety Scale (SCAS). All questionnaires were checked for data completeness and students were given an opportunity to complete any unanswered items. Parents were invited to complete an online survey which included demographic questions and standardised questionnaires pertaining to the child and family factors of interest.

### Measures

The psychometric properties of the questionnaires used to measure risk and protective factors and transition outcomes are provided in Online Resource 1.

#### Transition Outcomes

**Quality of Life.** QOL was assessed using the KIDSCREEN-27 (Ravens-Sieberer et al., 2007), a 27-item questionnaire for children and adolescents aged from 8 to 18. This measure was chosen for its simple and clear language and relative brevity. Students completed the Child and Adolescent (self-report) version. The KIDSCREEN has been recommended as a robust measure of QOL for children with ASD (Tavernor et al., 2013). It assesses five QOL domains however only the Total QOL score was used in this study.

**Student Mental Health.** Mental health was measured using the Strengths and Difficulties Questionnaire (SDQ) (Goodman, 2001). The SDQ is one of the most frequently used measures of mental health/psychopathology in school transition research. It consists of 25 items measuring hyperactivity/inattention, conduct problems, emotional symptoms, peer relationship problems, and prosocial behaviour for 3-16-year-olds.

**School Belonging.** School belonging was measured the Psychological Sense of School. Membership (PSSM) scale (Goodenow & Grady, 1993). This self-report questionnaire comprises 18 items which are measured on a 5-point Likert scale (1 = not at all true; 5 = completely).

#### Risk and Protective Factors

Risk and protective factors were drawn from a review of the wider school transition literature and included sociodemographic factors (age, gender, ethnicity, family SES), child factors (internalising and externalising problems, challenging behaviour, sleep problems, executive functioning and academic ability), family factors (family functioning and participation in family activities), and school factors (school SES, attendance, school refusal).

#### Sociodemographic Factors

**Family Socio-Economic Status (SES).** The Socio-Economic Indexes for Areas (SEIFA) is a general measure of relative disadvantage used by the Australian Bureau of Statistics. It is based on the residential postcode of the family and uses data from 2016 Census data such as income and education. SEIFA provides the average socio-economic characteristics of people living in those postcodes. The average SEIFA score is 1000 with a SD of 100. It is recommended that SEIFA deciles are used in research. The lowest scoring 10% of SEIFA scores are given the decile number 1 whereas the highest 10% of areas are given the decile number 10.

**Parental Education.** Family socio-economic status is often measured by a combination of parental education, income and employment status, however the strong association between parental education and school success warrants including this as a separate factor for analysis (Harding et al., 2015). Parents were asked to report their highest level of education (Year 10 or equivalent, Year 12 or equivalent, Certificate or Diploma, Bachelor’s Degree, or Post-Graduate Degree).

**Family Structure.** Parent/guardians were asked to describe their employment status, their family structure (who the student lives with), and the number of siblings the student had.

#### Child Factors

**Autism Spectrum Traits.** Autistic traits were assessed using the Social Responsiveness Scale 2nd edition (SRS-2) (Constantino & Gruber, 2012). This measure was chosen for its sound psychometric properties (Bruni, 2014), ease of use, and its utility for assessing autistic traits in students without a diagnosis of ASD. It produces a Total score that quantifies the level of severity of social deficits in the autism spectrum. Raw scores are converted into T scores for gender and respondent type. The questionnaire produces 5 subscales however only the Total score was used in this study.

**Anxiety.** Anxiety was assessed using the Spence Children’s Anxiety Scale© (SCAS) parent scale and child (self-report) scale (Spence, 1998). The total score is derived from summing all the items except the 6 positive fillers and six subscale scores can also be computed (Spence, 1998), however only the Total score was used in this study.

**ADHD Symptoms.** ADHD symptoms were assessed using the Strengths and Weaknesses of Attention-Deficit/Hyperactivity-symptoms and Normal-behaviours (SWAN) rating scale (Swanson et al., 2012). This measure was chosen for its dimensional nature (similar to the SRS-2) which enables measurement of strengths in attention and behavioural control as well as weaknesses. Parents were asked to rate their child on each item in comparison with children of the same age. Two subscales link to DSM-5 ADHD symptoms: an inattention subscale (9 items) and a hyperactivity/impulsivity subscale (9 items). Nine items relate to conduct problems. Higher scores on this measure indicate greater symptom severity.

**Sleep Problems.** Sleep problems were measured using the Children’s Sleep Habits Questionnaire (CSHQ; Owens, 2000). The scale provides a total sleep problem score as well as eight subscales (Owens et al., 2000), however only the Total Sleep Problem score was used in this study.

#### Family Factors

**Family Functioning.** Family functioning was assessed using the Family Adaptability and Cohesion Evaluation Scale 4th Edition (FACES-IV) (Olson, 2011). The scale was designed to measure two dimensions of family functioning: cohesion and flexibility. Cohesion was conceptualised in the circumplex model of family functioning as family closeness and emotional bonding. Flexibility referred to the consistency of roles, rules and expectations in the family and the ability to adjust to change (Olson, 2011). Each dimension could be adaptive or pathological depending on the degree of cohesion and flexibility. Balanced levels of cohesion were viewed as optimal for family functioning whereas very high levels of cohesion (termed enmeshment) and very low levels (termed disengagement) were considered unbalanced and problematic. Similarly, balanced levels of flexibility were viewed as optimal whereas very low levels of flexibility (termed rigidity) and very high levels (termed chaotic) were viewed as unbalanced and not conducive to healthy family functioning (Olson, 2000).

**Family Participation.** Family participation was measured using the Participation and Environment Measure for Children and Youth (PEM-CY) (Coster et al., 2012). This parent-report measure for young people aged 5 to 17 years assesses participation in activities within the home, school and community. It is appropriate to use with young people with and without disabilities. Ten items pertain to participation in the home environment and the parent/guardian was asked to rate how often their child participated in each activity over the past four months.

#### School Factors

**School Attendance and School Refusal.** The parent/guardian was asked about the student’s school attendance (number of days absent) and whether the student sometimes refused to go to school (and, if so, the reason given for school refusal).

**School Participation.** School participation was measured using the Participation and Environment Measure for Children and Youth (PEM-CY) (Coster et al., 2012). One item from the 5-item school scale (classroom activities) was excluded as it asked about the frequency of group work, classroom discussions and in-class assignments which would really require the classroom teacher’s response rather than a parent.

**School Sector and Level of Educational Advantage.** Schools in Australia can be categorised into three school sectors: Government, Catholic and Independent. The Index of Community Socio-Educational Advantage (ICSEA) provides an indication of the socio-educational background of students within the school (ACARA, 2013). A lower ICSEA score indicates that the students who go to that school have a lower educational advantage. The average ICSEA score is 1000 with a SD of 100.

#### Data Collection – Post-transition

Students and parents were sent email reminders with a link to the online post-transition surveys after students had completed their first term of secondary school. Student surveys included three questionnaires: the SDQ, KIDSCREEN 27 and the PSSM. Parent surveys included the SDQ (parent report) and KIDSCREEN 27 (parent report).

#### Data Cleaning

All study variables were examined for missing data, normality and outliers using statistic and graphical measures. QOL and school belonging were approximately normally distributed, however mental health problems (SDQ scores) for students without ASD did deviate significantly from normal with moderate to high positive skew. Little’s MCAR test and separate variance t-tests were non-significant indicating that the data were missing completely at random. Missing values were replaced with Expectation Maximisation. Normality of study variables was assessed using histograms and the Kolmogorov-Smirnov (K-S) test.

### Data Analysis

Given the large number of potential factors in relation to the sample size, bivariate correlations were performed to identify any significant associations between AS traits, risk and protective factors and transition outcomes (Online Resource 3). Factors were selected for inclusion in multiple regression analyses on the basis that they were significant at *p* < 0.05 in the bivariate analyses (Talbot & Massamba, 2019).

## Results

Hierarchical multiple regressions were performed for each transition outcome to identify significant predictors (see Online Resource 2).

### Transition Outcome 1: Quality of Life

AS traits were positively associated with improved QOL after transition. Gender and oppositional defiant behaviour (ODD) were the only significant child factors associated with parent reported QOL. Enmeshed and chaotic family functioning were significant family factors and school attendance was a significant school factor. When these factors were included in a hierarchical multiple regression analysis, only child factors significantly improved the model accounting for 24% of the variance in parent reported change in QOL. The only significant predictor when autistic traits were accounted for was gender. Female students experienced lower QOL after the transition than male students.

### Transition Outcome 2: Mental Health

AS traits were negatively associated with changes in mental health across the transition. This indicates that mental health was improving for students with higher AS traits. FSIQ, anxiety, sleep problems, inattention, hyperactivity, and ODD were significant child factors; disengaged, enmeshed, and chaotic family functioning, parent education, and family structure were significant family factors; school refusal and school participation were significant school factors. Autistic traits significantly predicted changes in parent reported mental health accounting for 24% of the variance in the dependent variable. When child factors were included in the model, it was significant, explaining a further 17% of the variance in mental health. The addition of family factors was also significant, explaining a further 27% of the variance in mental health changes across the transition. The inclusion of school factors, however, was not significant. Autistic traits, sleep problems, disengaged family functioning, parent education and family structure were significant predictors of changes in mental health problems after transition.

### Transition Outcome 3: School Belonging

AS traits significantly predicted changes in school belonging explaining 9% of the variance in the dependent variable. When child factors (Age and FSIQ) were included in the model, it was not significant. The inclusion of family factors was significant explaining a further 23% of the variance in school belonging. The addition of school factors was also significant, explaining a further 12% of the change in school belonging after school transition. In the final regression model, which explained 49% of the variance in school belonging, autistic traits, FSIQ, parent education, school attendance, and school refusal were all significant predictors of changes in school belonging.

## Discussion

Contrary to the hypothesised decline in transition outcomes after transition, autistic traits were associated with improved QOL, mental health, and school belonging. Autistic traits explained 12% of the variance in QOL, 24% of the variance in mental health and 9% of the variance in school belonging. To help understand these results, child, family and school factors were examined for their contribution to the variance in each transition outcome.

Once autistic traits were accounted for, the only significant predictor of QOL was gender. Gender is one of the more commonly investigated factors in the wider school transition literature, however the majority of transition studies focussing on autistic students have had largely male samples (Nuske et al., 2019), therefore less is known about the unique experience of female students making the transition to mainstream secondary school. Female students in this study, across all levels of autistic traits, demonstrated a decline in parent reported QOL after transition compared to male students. This finding supports research demonstrating that female adolescents reported lower QOL than males in general (Bisegger et al., 2005; Meade & Dowswell, 2016) and after the transition to secondary school (Simmons et al., 1987). One possible explanation for this finding is that female students in this study had much higher anxiety and higher anxiety predicts lower QOL (Brenes, 2007). Females have consistently been reported to have a greater propensity to developing an anxiety disorder (McLean et al., 2011) and this vulnerability is exacerbated by psychosocial stressors (Hallers-Haalboom et al., 2020). The transition to secondary school is one such psychosocial stressor as it involves relational instability as students experience the loss of support from close relationships formed at primary school and the need to establish new friendships and support networks (Symonds & Galton, 2014). This disruption of friendships and support may be particularly concerning for female students who, typically, have a greater need for affiliation than male students (Drescher & Schultheiss, 2016). The importance of the disruption of friendships to QOL for female autistic students during school transition is less clear. Although studies have noted that autistic females desire friendship in much the same way as non-autistic females, they are also more likely to have a smaller friendship circle and their friendships are often described as more intense (Sedgewick et al., 2019). Furthermore, autistic girls have greater difficulty dealing with conflict within their friendships (Sedgewick et al., 2019; Vine Foggo & Webster, 2017). During the early weeks of transition, there is often relational instability as students move between different social groups before finally settling into an established friendship group. This may be problematic for autistic girls who tend to find a few close friends and stick to them (Sedgewick et al., 2019). Other studies have reported autistic girls to be on be on the periphery of social circles, neither accepted nor rejected, but essentially ignored (Dean et al., 2014). Female autistic students are also reported to use camouflaging strategies to fit in (Dean et al., 2017) and while this may have social benefits, it can also negatively impact wellbeing (Tierney et al., 2016). Future studies examining gender differences in friendship quality and development for autistic students and how this impacts QOL during school transition would benefit from examining these factors with a larger sample and over a longer period.

The school transition domain of parent reported mental health was primarily predicted by family factors once autistic traits were accounted for. The only child factor that remained a significant predictor was sleep problems. Autistic traits were significantly associated with more parent reported sleep problems in this study and sleep problems predicted poorer mental health after autistic traits were accounted for. This corresponds to other research that sleep problems are more prevalent in autistic young people (Díaz-Román et al., 2018; Souders et al., 2017) and are associated with poorer mental health in both young people with autism (Cohen et al., 2014; May et al., 2015) and non-autistic young people (Hayes & Bainton, 2020). The impact of school transition on sleep or, conversely, the impact of pre-existing sleep problems on school transition outcomes is poorly understood (Chong et al., 2020). The current study’s finding that sleep problems significantly predict poorer mental health after transition is, therefore, an important addition to the literature.

Significant predictors of improved mental health after transition were students living with their mother and father, and mothers with higher educational qualifications, whereas disengaged family functioning was associated with poorer mental health after transition. Family structure has previously been found to be significantly associated with adolescent mental health and academic achievement (Barrett & Turner, 2005; Park & Lee, 2020), school attendance and health outcomes (Krueger et al., 2015). The majority of studies report that a child living with their mother and father is protective for mental health, aligning with the current study’s findings (Booysen et al., 2021). Two mechanisms that are conjectured to partially mediate this association are family SES and family functioning (Barrett & Turner, 2005e et al., 2012). In the current study, disengaged family functioning was associated with poorer mental health after transition. Disengaged functioning is considered to reflect unbalanced levels of family cohesion where family members have high levels of independence but low levels of warmth, affection, attachment and commitment to the family (Olson, 2011). Disengaged family functioning has previously been associated with poorer mental health of college students (Berryhill & Smith, 2021) and with poorer adjustment for students starting school (Sturge-Apple et al., 2010). The current finding underscores the importance of healthy family functioning during the transition to secondary school.

Parent education was the other significant predictor of changes in mental health after transition and was also the strongest predictor of improved school belonging after transition once autistic traits were accounted for. The association of higher maternal education^2^ with improved school outcomes is well documented and is thought to operate through several mechanisms such as more cognitively stimulating parenting practices, transmission of cultural capital, and negotiation and management of educational experiences (Harding et al., 2015). It is likely that some of these mechanisms also contribute to improvement in mental health and school belonging. For example, mothers who engage in cognitively stimulating experiences such as reading to their children and helping with homework and transmit cultural capital through frequent engagement in high culture activities such as art and music classes and museum visits (Jæger & Møllegaard, 2017) not only contribute to educational success but may also be contributing to their child’s mental health (Reiss et al., 2019). Similarly, it has been shown that highly educated mothers are more confident and skilled at navigating educational systems and effectively model for their children how to speak with teachers and how to interact with others at school in socially valued ways (Harding et al., 2015). Therefore, the transmission of cultural capital may facilitate the transition to secondary school and improve perceptions of school belonging.

The only significant child factor associated with improved school belonging after transition was cognitive functioning however the relationship was not in the expected direction. FSIQ scores were negatively associated with improved school belonging after the school transition. The relationship between IQ and academic achievement is well established (Deary et al., 2007; Roth et al., 2015), however the association between IQ and other school functioning indicators such as social-emotional functioning and school belonging is less clear (Allen & Kern, 2017). There is some suggestion that more cognitively able students may find the first year of secondary school boring as there can a great deal of revision of previous learning (Galton et al., 2000). Future research examining changes in school belonging after transition could further explore the influence of cognitive functioning.

School attendance was a significant predictor of school belonging when autistic traits were accounted for. School attendance has previously been linked to school belonging (Korpershoek et al., 2020) and is associated with many other important school outcomes (Gottfried, 2014; Hancock et al., 2013; Wood et al., 2012). Students with autistic traits are considered vulnerable to school attendance problems and this is particularly true for students attending mainstream schools (Totsika et al., 2020). There are various reasons for non-attendance, however school refusal behaviour (SRB) is the most commonly cited reason for autistic students (Totsika et al., 2020). Similarly, in the current study, autistic traits were associated with increased SRB and SRB was a significant predictor of poorer school belonging. The importance of monitoring school attendance across the transition to secondary school is important given findings that attendance declines in the first year of secondary school and declines thereafter (Hancock et al., 2013). Students with anxiety, ADHD, depression, and conduct disorder have lower school attendance rates (Hancock et al., 2013) as do students with ASD (Preece & Howley, 2018). The importance of consistent school attendance for positive wellbeing, social, and academic outcomes is well documented (Hancock et al., 2013), however the examination of school attendance for autistic students is not as well understood (Totsika et al., 2020).

SRB was a significant predictor of lower school belonging once autistic traits were accounted for. SRB is strongly associated with anxiety, particularly social anxiety (Gonzálvez et al., 2019). It has also been associated with being bullied (McClemont et al., 2020; Ochi et al., 2020), sleep problems (Hochadel et al., 2014), depression and challenging behaviour (Ingul & Nordahl, 2013), and poorer family functioning (Gonzálvez et al., 2019). SRB often has its onset in adolescence and is associated with many negative outcomes such as poor academic achievement, disconnection from peers, and family stress (Kearney, 2008). Early detection and management are considered vital before SRB becomes entrenched (Sewell, 2008), therefore families and schools should be alert to early signs of SRB during the transition to secondary school, particularly for autistic students.

### Study Limitations and Future Directions

Firstly, despite all primary schools in one regional area being invited to participate, only one of the participating schools had an Index of Community Socio-Educational Advantage (ICSEA) score below 1000. Similarly, only two secondary schools had an ICSEA score below 1000, indicating that the study did not adequately represent schools with lower socio-educational advantage. After the initial email invitation was sent, all schools who did not respond received a hard copy invitation along with a plain language statement and principal consent form. Only six schools (10%) formally declined and only four provided a reason for not participating. The reasons included being involved in other research, excessive workload, moving to a new school building, and “other priorities”. The reason for the paucity of low ICSEA schools in the study can only be surmised, however one staff member from a very disadvantaged school informally told the researcher that the staff were under a great deal of stress and that imposing any additional demands on the teachers was not encouraged. Consequently, students who participated in this study predominately attended schools with higher socio-educational advantage which may have contributed to their successful transition experience and academic progress. Numerous studies have attested to the importance of school-level educational advantage in advancing students’ academic progress and school engagement (Chesters, 2019; Chesters & Daly, 2018; Goss et al., 2016; Perry & McConney, 2010). Future transition studies should focus on recruitment to less advantaged schools to help clarify the effects of socio-educational advantage on transition outcomes.

A second limitation was that there were some factors potentially associated with school transition success that were not examined in this study. Although this study provided a broad coverage of child, family and school factors in the analysis, other factors often experienced by autistic students such as sensory issues, physical health issues such as gastrointestinal issues, and gender dysmorphia were not examined. A further limitation was that school transition indicators were only measured twice – in the final year of primary school and in the first term of secondary. It has been suggested that the transition period comprises the last year of primary school and the first two to three years of secondary school (Zendarski et al., 2016). Though few studies have employed longitudinal designs, the studies that have used multiple time points to examine transition outcomes have noted different trajectories over time (Benner & Graham, 2009). For most students, initial difficulties dissipated by the end of the first year of secondary school, however a minority experienced an escalation of difficulties (West et al., 2010). Although students with autistic traits reported improved QOL and school belonging after transition, this improvement may reflect a “honeymoon period” where students see their new school through “rose-tinted glasses” and the novelty factor is still fresh (Symonds & Galton, 2014). Future studies would benefit from examining these transition outcomes once a student has completed a year of secondary school as well as over multiple time points in their secondary education.

In conclusion, this study provided a valuable extension to the literature by examining school transition through a Life Course Theory lens. In addition to exploring how autistic traits in a school-based sample of students were associated with three indicators of transition success, it also provided a unique examination of how child, family and school factors explained additional variance in these transition outcomes. Gender was a significant predictor of QOL whereas variance in school belonging was predicted by cognitive functioning, parent education, attendance and school refusal. Changes in child mental health after transition were mainly explained by family factors including family structure, family functioning and parent education. Autistic traits and child sleep problems, however, were also significant predictors of mental health after transition.

## Footnotes

1 The authors acknowledge the debate about preferred language when referring to autistic people (see Kenny et al. 2016 for a valuable discussion on this topic) and use both person-first and identity-first language in this paper to honour both points of view.

2 ^2^ As all but 3 respondents were mothers, the ensuing discussion predominately refers to maternal education.
